# Hyperendemic dengue transmission and identification of a locally evolved DENV-3 lineage, Papua New Guinea 2007-2010

**DOI:** 10.1371/journal.pntd.0006254

**Published:** 2018-03-01

**Authors:** Dagwin Luang-Suarkia, Oriol Mitja, Timo Ernst, Shannon Bennett, Alfred Tay, Russell Hays, David W. Smith, Allison Imrie

**Affiliations:** 1 School of Biomedical Sciences, Faculty of Health and Medical Sciences, University of Western Australia, Nedlands, Western Australia, Australia; 2 Lihir Medical Centre, Lihir Island, Papua New Guinea; 3 Department of Microbiology, California Academy of Sciences, San Francisco, California, United States of America; 4 James Cook University, Cairns, Queensland, Australia; 5 Pathwest Laboratory Medicine WA, Nedlands, Western Australia, Australia; Oregon Health and Science University, UNITED STATES

## Abstract

**Background:**

Dengue is endemic in the Western Pacific and Oceania and the region reports more than 200,000 cases annually. Outbreaks of dengue and severe dengue occur regularly and movement of virus throughout the region has been reported. Disease surveillance systems, however, in many areas are not fully established and dengue incidence is underreported. Dengue epidemiology is likely least understood in Papua New Guinea (PNG), where the prototype DENV-2 strain New Guinea C was first isolated by Sabin in 1944 but where routine surveillance is not undertaken and little incidence and prevalence data is available.

**Methodology/Principal findings:**

Serum samples from individuals with recent acute febrile illness or with non-febrile conditions collected between 2007–2010 were tested for anti-DENV neutralizing antibody. Responses were predominantly multitypic and seroprevalence increased with age, a pattern indicative of endemic dengue. DENV-1, DENV-2 and DENV-3 genomes were detected by RT-PCR within a nine-month period and in several instances, two serotypes were identified in individuals sampled within a period of 10 days. Phylogenetic analysis of whole genome sequences identified a DENV-3 Genotype 1 lineage which had evolved on the northern coast of PNG which was likely exported to the western Pacific five years later, in addition to a DENV-2 Cosmopolitan Genotype lineage which had previously circulated in the region.

**Conclusions/Significance:**

We show that dengue is hyperendemic in PNG and identify an endemic, locally evolved lineage of DENV-3 that was associated with an outbreak of severe dengue in Pacific countries in subsequent years, although severe disease was not identified in PNG. Additional studies need to be undertaken to understand dengue epidemiology and burden of disease in PNG.

## Author summary

Dengue virus (DENV) was first identified in Papua New Guinea (PNG) in 1944. Dengue is currently assumed to be an endemic disease in PNG although there is little incidence or prevalence data, and the evidence consensus for dengue presence is low. Routine surveillance is not undertaken and dengue is not a notifiable disease. Severe dengue is rarely identified by local clinicians and the reasons for this are unclear but may be related to poor recognition of dengue and a low index of suspicion, despite high incidence and prevalence rates in neighbouring countries. For example, Indonesia shares borders with PNG and regularly reports outbreaks of severe dengue and transmission of multiple DENV serotypes. DENV infection is identified in travellers from PNG however there are no data on locally circulating strains and how they may compare to viruses associated with severe dengue epidemics in other countries in the Asia Pacific region. We identified evidence for previous infection with all four DENV serotypes among people living on the northern coast of PNG, in Madang, and on Lihir Island in the Bismarck Archipelago off the northeastern coast. We also detected DENV-1, DENV-2, and DENV-3 virus in febrile patients, and we describe the first whole genome sequences of endemically circulating DENV since the prototype 1944 DENV-2_New Guinea C_ strain was characterized. Of note, severe dengue was not diagnosed in any patient infected with these viruses in PNG although introduction of the PNG DENV-3 strain into the Solomon Islands five years later resulted in a large outbreak of severe dengue with hospitalizations and deaths in that country. Dengue epidemiology and burden of disease should be investigated in PNG.

## Introduction

The dengue viruses (DENV) are the most important arboviral pathogens of humans causing an estimated 390 million infections annually, of which approximately one quarter are symptomatic [1]. Infection with DENV causes a spectrum of clinical outcomes ranging from self-limiting febrile illness (dengue fever, DF) to potentially fatal severe dengue, characterized by plasma leakage, thrombocytopenia, and hypovolemic shock. Dengue is endemic in more than 100 tropical and subtropical countries, where the principal mosquito vectors *Aedes aegypti* and *Aedes albopictus* are found [2, 3].

DENV is a single-stranded positive-sense RNA virus of the *Flaviviridae* family. Like other RNA viruses, DENV displays considerable genetic diversity and is grouped into four antigenically distinct serotypes (DENV-1-DENV-4) which may be distinguished on the basis of serum neutralization tests. The four serotypes are more precisely classified, using phylogenetic approaches, into distinct genotypes which have been defined as clusters with nucleotide sequence divergence of not more than 6% [4]; lineages within the genotypes may represent strains with similar geographic origins [5]. Certain genotypes have been associated with more [6,7] or less [8] virulent disease, and there is some evidence for humoral and cellular immune selection focused on viral B- and T-cell epitopes [9,10]. DENV genetic diversity thus appears to impact host mechanisms shown to mediate pathogenesis [11] and ultimately, disease severity.

Dengue was first identified in Papua New Guinea (PNG) when Sabin isolated the prototype DENV-2_New Guinea C_ strain from febrile soldiers deployed on the northern coast of New Guinea in 1944 [12]. A DENV strain with similar biologic features as the prototype DENV-1_Hawaii_ that Sabin had recently isolated from febrile soldiers in Hawaii, was also isolated from soldiers in the same area of New Guinea in 1944 [13], suggesting that at least two serotypes may have circulated in the north of PNG in the 1940s. More recently, DENV-1-3 genetic data derived from viremic travellers returning to northern Australia from PNG between 1999–2010 [14] indicate that dengue is endemic in PNG, supporting an earlier serological study demonstrating a seroprevalence rate of 8% among patients presenting to clinics in Madang with acute febrile illness [15]. Despite the likely transmission of multiple DENV serotypes and the potential for introduction of DENV from endemic neighbouring countries which experience large-scale epidemics of severe dengue [16], little is known about the epidemiology and transmission dynamics of dengue in PNG, where dengue surveillance is not undertaken, individuals with acute febrile illness are not routinely tested for DENV infection, and where severe disease is rarely reported.

We sought to determine DENV serotype and genotype prevalence in local populations presenting with febrile illness, or with a range of non-febrile conditions. We sequenced whole genomes of DENV and conducted a phylogenetic analysis to determine the evolutionary origin of PNG DENV. In addition we determined anti-DENV-1-4 neutralizing antibody profiles in adults and children in order to assess prevalence and serotype diversity.

## Materials and methods

### Study area

Samples analysed in this study were collected from Madang, on the northern coast of PNG, and from Lihir Island in New Ireland Province. Madang is a town of about 30,000 people with a sea port that is a major hub for domestic and international shipping, and an airport where domestic flights from throughout PNG arrive several times each day. Lihir Island is 800 kilometres northeast of Madang in the Bismarck Archipelago, in the western Pacific Ocean. The island’s population doubled to more than 12,000 people after establishment of a gold mine in 1997 and although many residents still live a predominantly traditional subsistence lifestyle, in recent years there has been an influx of PNG-national and expatriate mine workers and development of an international airport and sea port.

### Patients and samples

Madang sera were collected from febrile patients presenting to the outpatient clinic of Yagaum rural hospital or to Jomba town clinic from September 2007 through June 2008, and who were enrolled in a malaria study [15]. Sera excluded for malaria antigens were tested for anti-DENV IgG and IgM, and NS1 antigen; 8% (46/578) were identified as probable acute DENV infection ie. NS1 antigen-positive and/or anti-DENV IgM-positive. A total of 55 acute phase sera (46 sera identified serologically as probable acute DENV infection plus 9 additional febrile sera that were not tested for DENV), were assessed in the present study for the presence of DENV by DENV E gene RT-PCR and virus isolation was attempted on RT-PCR positive samples. Whole genomes of isolated viruses were sequenced using Illumina. Convalescent sera from 119 patients excluded for acute DENV infection and who presented for recollection were collected an average of 29.6 days (range 5–159 days) after the first patient visit and tested for the presence of anti-DENV neutralizing antibody (NAb) to all four serotypes simultaneously using a microneutralization assay optimized for small sample volumes [17].

Lihir sera (55 in total) were collected from patients presenting to the outpatient clinic of Lihir rural health centre from May through November 2010 during pre-employment medical visits, at antenatal screening visits, or from patients presenting for a range of conditions including joint pain, diabetes and fever. The sera were also assessed for DENV genomes (11/55 sera from febrile patients) and for anti-DENV NAb (44/55 sea from patients with non-febrile conditions).

Patient data are summarized in Table 1.

**Table 1:** Patient clinical and demographic data.

Category	Location	Samples collected	No. sera	Age Median (range)	Sex		
					M	F	NS*
Acute DENV infection	Madang	September 2007 –June 2008	55	3 (0.5–50)	26	24	5
	Lihir	May-November 2010	11	39.5 (32–47)	1	1	9
Convalescent/non-infectious/non-febrile	Madang	October 2007 –July 2008	119	14.2 (0.5–60)	53	66	
	Lihir	September-October 2010	44	25.5 (12–49)	5	39	

* Not Specified

### Ethical considerations

Ethics approval for this study was granted by the Medical Research Advisory Committee, Ministry of Health, Government of Papua New Guinea (2010) and the Human Research Ethics Committee, University of Western Australia (2010). All data analysed were anonymized.

### Virus isolation

DENV was isolated from serum by inoculation onto monolayers of Vero cells [5]. Briefly, 100μl of acute phase serum was inoculated onto a Vero cell monolayer in minimal media in a 2.5 ml culture tube, and incubated overnight. On the following day the inoculum was removed and 3ml of DMEM with 2% FBS (supplemented with L-glutamine and antibiotics) was added to the cells, and the culture was maintained at 37°C with 5% CO_2_ for 7 days or until cytopathic effect (CPE) was observed; for most samples a blind passage into a second 2.5 ml culture tube was required in sorder to isolate virus. Successful virus isolation was identified by NS1 antigen ELISA (Platelia Dengue NS1 Antigen ELISA; Bio-Rad, Australia).

### RNA extraction and RT-PCR

Viral RNA was extracted from 140 μl of culture supernatant using QIAmp viral RNA Mini kits (Qiagen), according to the manufacturer’s instructions. cDNA was synthesized from extracted RNA using SuperScript III First-Strand Synthesis System for RT-PCR (Invitrogen) as per the manufacturer’s instructions. DENV serotype was identified by RT-PCR using serotype-specific primers [5], and the LongRange PCR Kit (Qiagen) (thermocycling conditions are available on request).

### Whole genome sequence analysis

#### RNA extraction and library preparation

DENV NS1-positive cultures were expanded by passage in 25 ml tissue culture flasks in a volume of 15 ml. Supernatants were harvested and clarified by centrifugation at 1400 rpm for 10 minutes at 4 deg C to remove cellular debris. The clarified supernatant was then transferred to a Millipore centrifugal filter unit (100,000kDa) and centrifuged at 4000 x g for 20 minutes. RNA was extracted from 150–200 μl concentrated virus supernatant using the Roche High Pure RNA isolation kit as per the manufacturer’s instructions. RNA mass/concentration was then determined by fluorescent detection using the Qubit system with Qubit RNA HS Assay kit (Invitrogen). All RNA was stored at -80°C until used. DNA libraries were prepared with the TruSeq Stranded mRNA kit (Illumina), with the use of 200ng of total starting RNA and the exclusion of the polyA selection step. The DNA library sizes were validated with a High Sensitivity DNA kit (Agilent) on the Agilent 2100 Bioanalyzer. The concentration of the libraries were normalised to 4nM using HT1 buffer supplied by Illumina and pooled together. The final concentration of the pooled DNA library was then re-examined using the Qubit ssDNA Assay kit (Invitrogen). The pooled DNA library was sequenced using a MiSeq Reagent Nano Kit, v2 (Illumina).

#### Nucleic acid sequencing and phylogenetic analysis

Sequences were trimmed, quality checked and assembled against DENV reference genomes into contigs using CLC Genomics Workbench (Ver. 8.5.1). Complete assembled contigs were checked for chimeras and aligned against publicly available sequences using MACSE [18]. Alignments were verified in Se-Al 2.0 (http://tree.bio.ed.ac.uk/software/seal/) and analyzed using RAxML to generate a maximum-likelihood (ML) tree with ML bootstrap support based on the optimal number of ML bootstrap replicates [19], using a general-time-reversible substitution model with among-site rate heterogeneity accounted for by the gamma model (GTR + gamma, best fit as indicated in datamonkey.org). Trees were mid-point rooted. To identify nucleotide substitutions that have occurred on the internal branches of lineages formed during the evolution of DENV, particularly in PNG, we mapped changes onto the ML phylogenetic tree using a parsimony-based method implemented in MacClade 4.08 [20].

### Microneutralisation assay

A serum microneutralization (MN) assay was used to measure serum anti-DENV antibodies [17]. This approach was selected to allow simultaneous assessment of antibody to all four DENV serotypes in samples with limited volumes. Standard anti-DENV-1-4 sera NIBSC 05/248 (National Institute for Biological Standards and Control [NIBSC], Potter’s Bar, Hertfordshire, United Kingdom) were assayed against the homologous DENV prototype strains DENV-1_Hawaii2001_; DENV-2_NGC;_ DENV-3_H-87_ and DENV-4_H-241_ and consistently produced MN titres of 10–20 and thus, the cut-off value for a positive test result was a reciprocal serum dilution of 10. Subject results were summarized and presented as geometric mean titres (GMT). Cross-neutralization experiments in which Standard DENV-1-4 sera were each tested against heterologous prototype DENV consistently produced negative results. Standard anti-JE serum (NIBSC 02/182) and serum samples from individuals with diagnosed other flavivirus infection (JEV, MVEV, and KUNJV) were tested against DENV-1-4 and were always negative.

### Accession numbers

All sequences have been deposited in GenBank and assigned accession numbers KY794785-KY794790.

## Results

### Transmission of multiple DENV serotypes, PNG

Three circulating DENV serotypes: DENV-1, DENV-2 and DENV-3 were identified by full length E gene PCR of acute phase serum samples from 17 febrile patients sampled in Madang between 2007–2008 and Lihir in 2010 (Table 2).

**Table 2:** DENV identified in febrile patients, PNG.

Sample	Sex, Age (Years)	Location	Date collected	DENV Serotype	Strain ID	GenBank Accession No.
1	M, 9	Madang	26-Sept-2007	1	-	-
2	F, 21	Madang	05-Oct-2007	3	-	-
3	F, 29	Madang	9-Oct-2007	3	D3_PNG_Madang_071009_2007	KY794787
4	F, 9	Madang	19-Dec-2007	2	-	-
5	F, <1	Madang	4-Feb-2008	1	-	-
6	NA, <1	Madang	6-Feb-2008	2	-	-
7	F, 0.6	Madang	11-Feb-2008	2	-	-
8	M, <1	Madang	24-Apr-2008	3	D3_PNG_Madang_080424_2008	KY794788
9	M, 3	Madang	25-April-2008	2	-	-
10	F, 9	Madang	6-May-2008	2	-	-
11	M, 0.5	Madang	14-May-2008	2	-	-
12	F, 4	Madang	21-May-2008	3	D3_PNG_Madang_080521_2008	KY794789
13	M, <1	Madang	26-May-2008	2	-	-
14	F, 1	Madang	6-June-2008	2	-	-
15	M, 3	Madang	6-Jun-2008	3	D3_PNG_Madang_080606_2008	KY794790
16	M, 47	Lihir	11-May-2010	2	D2_PNG_Lihir_100511_2010	KY794785
17	F, 32	Lihir	27-Oct-2010	3	D3_PNG_Lihir_101027_2010	KY794786

DENV-1, DENV-2, and DENV-3 infections were identified in adults and children from Madang town or from villages and rural settlements around the town. In several instances, two serotypes were identified in individuals sampled within a period of 10 days. Two DENV cases originated in Lihir in May and October 2010 –a 47 year-old male Australian traveller infected with DENV-2 and 32 year-old female resident of Lihir infected with DENV-3. These data clearly identify hyperendemic DENV transmission on the northern coast of PNG, in Madang, and on Lihir Island in the Bismarck Archipaleago.

### Genome analysis identifies an endemic DENV-3 lineage that has evolved locally

**Fig 1:**
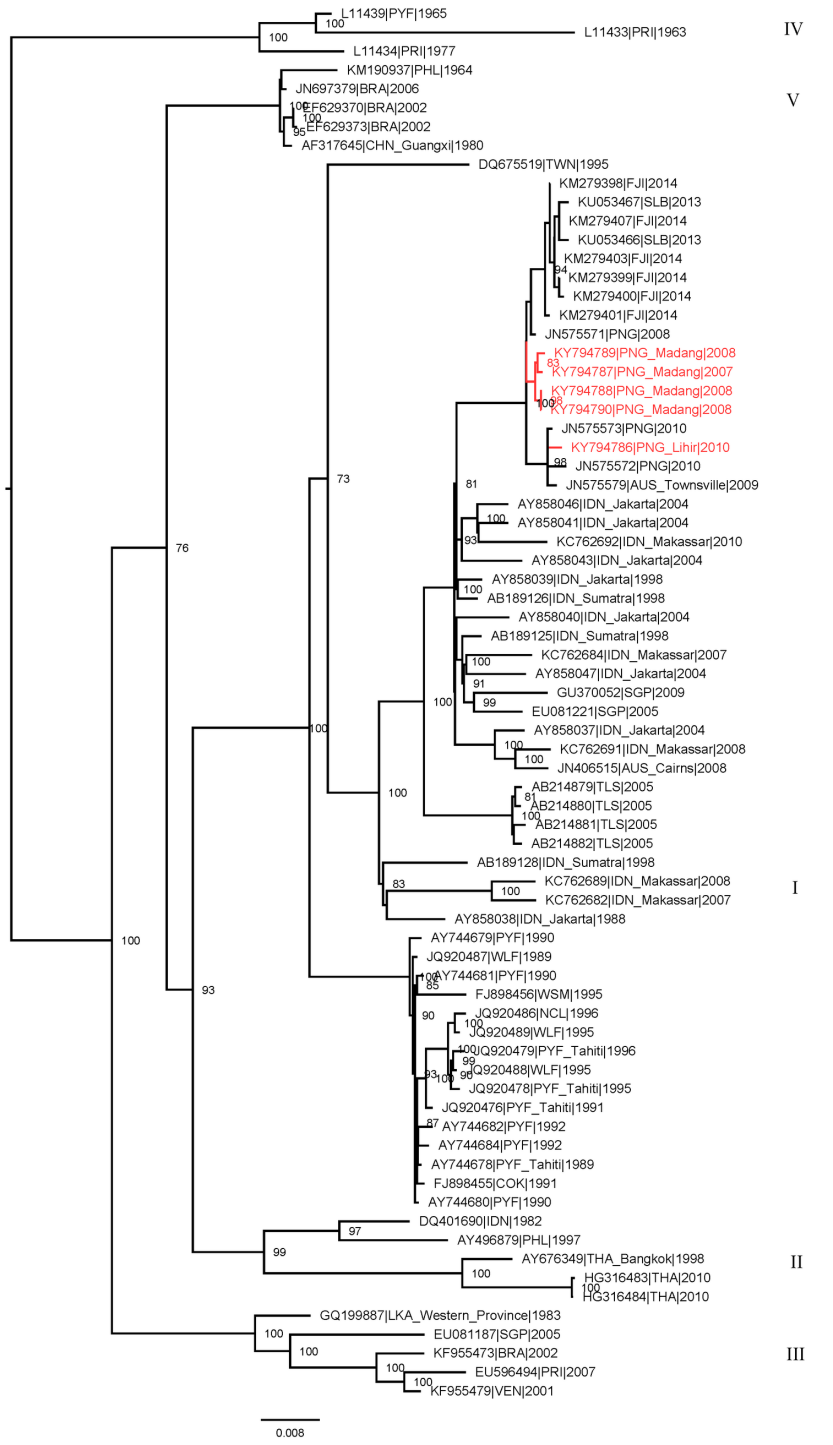
Phylogenetic tree of DENV-3 from Madang (2007–2008) and Lihir (2010). PNG DENV-3 (indicated in red) compared with reference viruses obtained from GenBank. The PNG viruses grouped with genotype 1 of DENV-3. Viruses isolated from Madang clustered together and were distinct from later strains, clustering with the Lihir isolate, identified in travellers to northern Australia originating in PNG. The tree was derived by maximum likelihood methods using whole genome sequences. Most reference virus sequences were complete envelope gene. Bootstrap support values are shown at nodes.

Whole genomes were sequenced from one DENV-2 and five DENV-3 isolates. Phylogenetic analysis of the five DENV-3 isolates indicated that the four from Madang group together to form a previously unidentified lineage within Genotype I (Fig 1). The Madang lineage included other DENV-3 from the region, including a group exported to Solomon Islands and Fiji and collected in 2013 [21] and 2014, respectively. The DENV-3 strain originating in Lihir in 2010 also grouped within Genotype I and clustered with a lineage formed by DENV-3 first found in travellers between PNG and northern Australia [14]. Collectively, these data identify a lineage of DENV-3 endemic to the northern coast of PNG, closely related to DENV-3 circulating in neighbouring Indonesia, and the subsequent introduction of this lineage into the western and south Pacific. The entire lineage is well supported and distinguished from other viruses within Genotype I by seven amino acid substitutions spanning several genes [prM, E, NS2A, NS3, NS5] (Table 3) and are indicated by the highlighted branch in Figure I. Several of these substitutions were non-conservative, involving the replacement of one encoded amino acid with another of very different properties. At least two of these substitutions were fixed through the process of positive selection according to at least one statistical test implemented in HyPhy [22] and the online version Data Monkey (http://www.datamonkey.org/ [23, 24].

**Table 3:** Substitutions along the DENV-3 PNG lineage formation.

Substitution	Gene Region	First Type	Replacement	Change
F242L	membrane	Phenylalanine; large (MW 165), nonpolar, aromatic	Leucine; MW 131, nonpolar, aliphatic	Nonconservative
L404S	envelope	Leucine; MW 131, nonpolar	Serine; MW 105, polar, phosphorylated, O-glycosylated	Nonconservative
V1305M^1^	NS2A	Valine; MW 117, nonpolar	Methionine; MW 149, nonpolar	Nonconservative
T1592I	NS3	Threonine; MW 119, polar, phosphorylated, O-glycosylated	Isoleucine; MW 131, nonpolar	Nonconservative
T1866S	NS3	Threonine; MW 119, polar, phosphorylated, O-glycosylated	Serine; MW 105, polar, phosphorylated, O-glycosylated	Conservative
A2021S	NS3	Alanine; MW 89, nonpolar	Serine; MW 105, polar, phosphorylated, O-glycosylated	Nonconservative
F3257Y^2^	NS5	Phenylalanine; large (MW 165), nonpolar, aromatic	Tyrosine; MW 181, polar, phosphorylated, O-glycosylated	Nonconservative

^1^ under positive selection according to the FEL, iFEL, and Toggle tests for selection

^2^ under positive selection according to the Toggle test for selection

**Fig 2:**
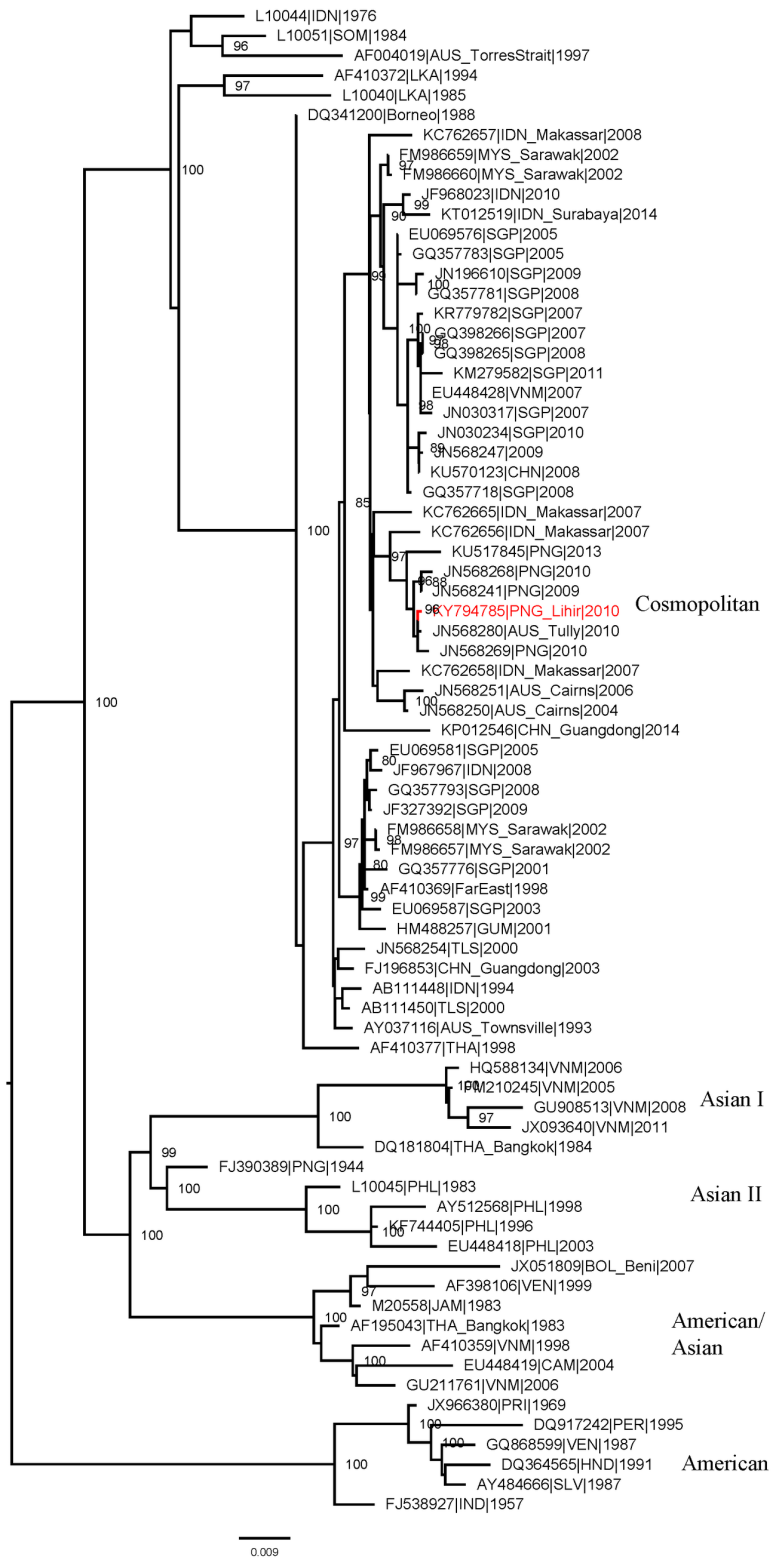
Phylogenetic tree of DENV-2 from Lihir, October 2010. PNG DENV-2 (indicated in red) compared with reference viruses obtained from GenBank. The Lihir virus grouped with the Cosmopolitan genotype of DENV-2, and clustered with DENV-2 identified in travellers entering northern Australia from PNG 2009–2010. The tree was derived by maximum likelihood methods using whole genome sequences. Most reference virus sequences were complete envelope gene. Bootstrap support values are shown at nodes.

The Lihir 2010 DENV-2 virus grouped with the Cosmopolitan genotype (Fig 2) and clustered with DENV-2 originating in Makassar, Indonesia in 2007 [25], and identified in travellers between PNG and northern Australia [14]. Cosmopolitan DENV-2 circulates widely in South and Southeast Asia, Africa, the Middle East, and northern Australia, and these data illustrate introduction of DENV-2 into the Western Pacific region from Southeast Asia.

### Previous DENV infection

**Fig 3:**
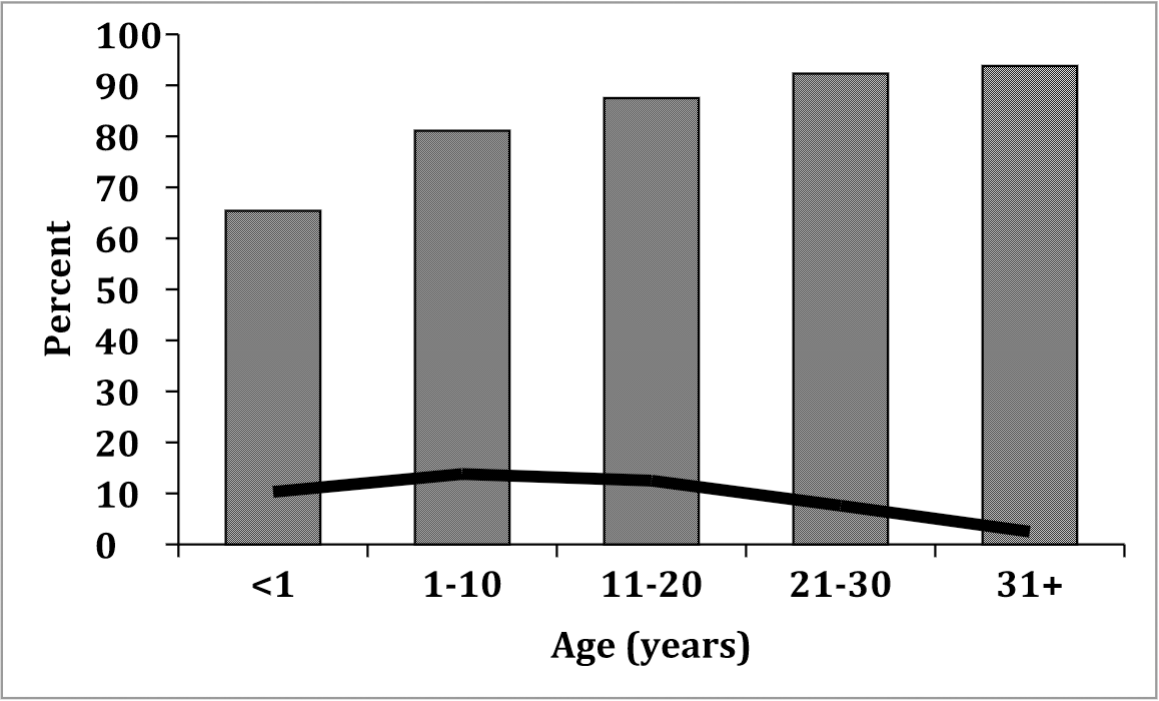
Dengue seroprevalence in PNG. Columns represent age groups, and the proportion of NAb-positive sera is shown for each group. Seroprevalence increases with age, plateauing after adults reach 20 years of age. Monotypic infections (black line) decline to close to zero with increasing age.

Overall dengue seroprevalence at the two study sites was 85.3%. Assessment of convalescent sera from residents of Madang identified previous DENV infection in the great majority of samples tested (101/119 sera; 84.9%), most of which showed multitypic NAb responses to all four DENV serotypes (Table 4). DENV-2 and DENV-3 GMTs were of greatest magnitude. Lihir sera showed a similar profile (Table 5) to that seen in Madang where the majority of sera were seropositive (38/44; 86.4%), multitypic DENV-1-DENV-4 NAb responses predominated and a majority neutralized all four serotypes. In both locations, monotypic NAb responses were identified in 11% of sera and overall were directed against each of the four serotypes. Seroprevalence increased with age (Table 6, Fig 3), a pattern that is typically seen in dengue-endemic areas.

**Table 4:** Anti-DENV neutralizing antibody profiles, Madang.

Anti-DENV NAb-positive		Anti-DENV NAb Titer*			
Serotype(s)	No. positive (%)	DENV-1	DENV-2	DENV-3	DENV-4
DENV-1	1 (1)	10	-	-	-
DENV-2	7 (6.9)	-	14.9(10–40)	-	-
DENV-3	1 (1)	-	-	320	-
DENV-4	2 (2)	-	-	-	10(10)
*Sub-total*, *monotypic^*	*11 (10*.*9)*				
DENV-1,-2	2 (2)	20 (20)	20 (20)	-	-
DENV-2,-3	6 (5.9)	-	26.9 (10–80)	40 (10–320)	-
DENV-2,-4	2 (2)	-	20 (20)	20 (10–40)	-
DENV-3,-4	2 (2)	-	-	20 (10–40)	10 (10)
DENV-1,-2,-3	2 (2)	113.1 (40–320)	20 (10–40)	28.3 (20–40)	-
DENV-1,-2,-4	6 (5.9)	17.8 (10–40)	25.2 (10–80)	-	15.9 (10–40)
DENV-1,-3,-4	5 (5)	13.2 (10–20)	-	60.6 (10–320)	30.3 (20–40)
DENV-2,-3,-4	4 (4)	-	14.1 (10–20)	20 (10–80)	14.1 (10–40)
DENV-1,-2,-3,-4	61 (60.4)	45.4 (10–640)	83.8 (10–640)	80.9 (10–640)	63.6 (10–640)
*Sub-total*, *multiple*	*90 (89*.*1)*				
Total	101 (100)				
Overall GMT		37.1(10–640)	61.5(10–640)	72.8(10–640)	46.4(10–640)

* NAb expressed as geometric mean titer (range)

^ Monotypic responses were largely in children (median age, 2 years)

- denotes titer <10

**Table 5:** Anti-DENV neutralizing antibody profiles, Lihir.

Anti-DENV NAb-positive		Anti-DENV NAb Titer *			
Serotype(s)	No. positive (%)	DENV-1	DENV-2	DENV-3	DENV-4
DENV-1	2 (5.4)	10	-	-	-
DENV-2	1 (2.7)	-	10	-	-
DENV-3	1 (2.7)	-	-	10	-
DENV-4	0 (0)	-	-	-	-
*Sub-total*, *monotypic*	*4 (10*.*5)*				
DENV-1,-2	2 (5.3)	20	20 (20–160)	-	-
DENV-1,-4	1 (2.7)	20	-	-	10
DENV-2,-4	2 (5.3)	-	20 (10–40)	-	10
DENV-1,-2,-3	3 (7.9)	40 (10–160)	127 (80–320)	15.9 (10–20)	-
DENV-2,-3,-4	4 (10.5)	-	33.6 (10–80)	11.9 (10–20)	10
DENV-1,-2,-3,-4	22 (59.5)	25.7 (10–640)	49.9 (10–640)	30.1 (10–160)	22.7 (10–640)
*Sub-total*, *multiple*	*34 (91*.*9)*				
Total	38 (100)				
Overall GMT		26.3(10–640)	49.4(10–640)	24.8(10–160)	18.6 (10–160)

* NAb expressed as geometric mean titer (range)

- denotes titer <10

**Table 6:** Age and sex distribution for seropositive individuals.

Age (years)	Frequency anti-DENV NAb-positive					
	Madang					
	Male, n = 53		Female, n = 66		Total, n = 119	
	No. sera	No. positive (%)	No. sera	No. positive (%)	No. sera	No. positive (%)
<1	10	7 (70)	16	10 (62.5)	26	17 (65.4)
1–10	15	12 (80)	22	18 (81.8)	37	30 (81.1
11–20	9	8 (88.9)	12	11 (91.7)	21	19 (90.5)
21–30	10	10 (100)	7	7 (100)	17	17 (100)
31+	8	8 (100)	9	9 (100)	17	17 (100)
NS	1	1 (100)			1	1 (100)
Total, Madang	53	46 (86.8)	66	55 (83.3)	119	101 (84.9)
	Lihir Island					
	Male, n = 4		Female, n = 40		Total, n = 44	
	No. sera	No. positive (%)	No. sera	No. positive (%)	No. sera	No. positive (%)
10–20	1	0(0)	2	2(100)	3	2 (66.7)
21–30	0	0 (0)	22	19 (86.4)	22	19 (86.4)
30+	2	2 (100)	13	11 (84.6)	15	13 (86.7)
NS	1	1 (100)	3	3 (100)	4	4 (100)
Total, Lihir	4	2 (50)	40	35 (87.5)	44	38 (86.4)

## Discussion

Although dengue was first described in Papua New Guinea more than 70 years ago when Sabin isolated the prototype DENV-2 strain New Guinea-C from US soldiers deployed along the northern PNG coast during the Second World War [12], the lack of reported case data and DENV transmission data since that time has meant that the distribution of dengue in PNG is not understood. In this study we identified infection with three DENV serotypes among febrile patients presenting to health clinics on the northern coast of PNG, in Madang, over a nine month period in 2007–2008, and infection with two serotypes within 6 months in 2010, in Lihir Island. These findings are consistent with hyperendemic DENV transmission between 2007–2010 and confirm that dengue is endemic in this country.

Our understanding of DENV transmission in PNG has previously been largely limited to detection of DENV in febrile travellers to northern Australia [14]. The Madang DENV-3 Genotype 1 lineage evolved locally and circulated over the year the study was conducted. Our phylogenetic analysis showed that it was most closely related to DENV-3 detected 3.5 years later in the Solomon Islands in January 2103 and then in the Fiji Islands in 2014, likely exported by travellers and having moved over distances of thousands of kilometres. PNG shares geographic borders with Indonesia, where dengue epidemics occur regularly and endemic, locally evolved DENV-3 lineages have been described [5, 25]. The Madang lineage is most closely related to DENV-3 originating in Indonesia, as is the DENV-3 originating in Lihir Island which clustered with DENV-3 identified in travellers to northern Australia.

Similarly, the DENV-2 virus identified in this study in a local resident of Lihir Island in 2010 is representative of strains known to circulate in the region, clustering with a highly similar strain originating in Makassar, Indonesia, in 2007; both viruses belong to a DENV-2 (Cosmopolitan genotype) clade also circulating in neighbouring Singapore in 2008 and identified in travellers to northern Australia in 2004 and 2006. Our results provide further evidence of DENV movement between endemic countries in the Asia Pacific region that has been described by ourselves and others [26–29], and also illustrate the value of sequence data as a means of understanding virus dispersal. PNG may be a source, or at least a place of transit, for dengue to enter the Pacific and further disseminate to other Pacific Island nations.

DENV epidemic virulence has been linked to introduction and transmission of specific serotypes and lineages [6,7]. The DENV-3 lineage we identified to be circulating in Madang in 2007–2008 and which our analysis showed to have evolved locally was subsequently associated with hospitalizations and deaths when it was introduced into the Solomon Islands in 2013 [30], although severe disease was not identified among patients in Madang. This entire lineage was distinguished by several amino acid substitutions that may influence virus phenotypic characteristics and ultimately, disease outcome. Severe dengue has also been linked to genetic polymorphisms including HLA type [31]; PNG and the Solomon Islands both belong to the Melanesian subgroup of Pacific Islanders and although relatively few HLA data are available for this population group, certain allele frequencies are known to be shared. Studies to assess the immunopathogenesis of dengue in PNG would be informative and should be undertaken in future.

Dengue seroprevalence was very high with an overall rate of 85.3%, and increased with age. More than half of seropositive individuals from Madang were greater than 11 years of age and about 40% were older than 20 years. This age distribution reflects opportunities for multiple exposures over the lifetime of the individual in a setting where dengue is endemic or is regularly introduced and indeed, anti-DENV neutralising antibody profiles were predominantly multitypic. A majority of individuals in Madang and in Lihir demonstrated responses to two or more DENV serotypes and most sera neutralized all four DENV serotypes. Serum samples included in this analysis were from two main groups–convalescent patients excluded for acute DENV infection in Madang, and clinic attendees in Lihir (predominantly women attending antenatal clinics) therefore these neutralization data likely reflect multiple DENV infections in the years prior to sampling, and corroborate the genetic evidence for circulation of multiple serotypes. Studies in rural Haiti and Nicaragua [32, 33] have shown that in endemic populations anti-DENV antibody prevalence increases with age and begins to plateau in adolescence reflecting long-term exposure to, and infection with, endemically circulating DENV. In the present study further support for endemic DENV transmission in PNG is indicated by the young age (median 2 years) of individuals in Madang with monotypic anti-DENV NAb responses to DENV-1, DENV-2, DENV-3 or DENV-4 whereas the majority of multitypic infections were in adults. Three individuals with monotypic NAb profiles were babies less than 1 year old and anti-DENV NAb were likely passively transferred maternal antibody. We do not know the age of the mothers but it is possible they were relatively young and/or had experienced a single DENV infection in the past, or that they had experienced multiple infections but that only antibodies to a single serotype were present at sufficiently high levels in the infant to be detectable in our assay.

Hyperendemic DENV transmission is associated with symptomatic dengue infection and with greater incidence of severe dengue [3]. No febrile patients were diagnosed with severe dengue in Madang [15] or Lihir and indeed, severe dengue is rarely identified in PNG despite the hyperendemic transmission we have identified and which has likely been occurring for a significant period of time. In previous studies we demonstrated high seroprevalence and predominantly multitypic NAb responses to all four serotypes in sera collected in New Guinea between 1959–1963 [17] indicating DENV transmission in the decades prior to sampling. The reasons for the rarity of severe disease are unclear but may be related to poor recognition of dengue and the lack of routine dengue surveillance. It is also possible that undefined DENV-specific immune mechanisms may contribute to the apparent rarity of severe dengue disease.

In summary we have identified hyperendemic dengue transmission in PNG in the period up to 2010, which has likely been occurring for many years. We also demonstrate circulation of DENV which has evolved locally, and shown by others to have been introduced into the western and south Pacific in subsequent years. The absence of regular sampling for DENV in PNG and the potential for misdiagnosis of febrile individuals has meant that the evidence consensus for dengue presence is low [2] and the true burden of disease is unknown. A dengue outbreak in Port Moresby was confirmed by the PNG Department of Health in 2016 [34] highlighting the need for additional studies to be undertaken to understand the epidemiology and impact of dengue in this country. An important aspect of this is to understand the origin and transmission patterns of PNG DENV, define endemic and introduced genotypes and lineages, and to characterize epidemic virulence associated with circulation of these viruses among the PNG population and within the Asia Pacific region.
